# IL-6 Transsignaling in Patients with Chronic Spontaneous Urticaria

**DOI:** 10.1371/journal.pone.0145751

**Published:** 2015-12-23

**Authors:** Alicja Kasperska-Zajac, Alicja Grzanka, Aleksandra Damasiewicz-Bodzek

**Affiliations:** 1 Department of Internal Diseases, Dermatology and Allergology in Zabrze, SMDZ in Zabrze, Medical University of Silesia in Katowice, Poland; 2 Department of Chemistry in Zabrze, Medical University of Silesia in Katowice, SMDZ in Zabrze, Poland; Northwestern University Feinberg School of Medicine, UNITED STATES

## Abstract

**Background:**

IL-6 trans-signaling is critically involved in the initiation and promotion of inflammatory and autoimmune diseases. Therefore, we investigated the clinical relevance of soluble members of IL-6 trans-signaling system in chronic spontaneous urticaria (CSU).

**Methods:**

IL-6, interleukin 6 soluble receptor (IL-6 sR) and soluble gp130 (sgp130) were measured by ELISA method in plasma from CSU patients and the healthy subjects. The data were related to activation of the acute phase response as indicated by serum C-reactive protein (CRP) concentration and compared between patients stratified by the disease activity.

**Results:**

Concentrations of IL-6, IL-6 sR, sgp130 in plasma and CRP in serum were significantly elevated in CSU patients compared with the healthy controls. CRP correlated significantly with IL-6 and sgp130, similarly IL-6 correlated significantly with sgp130. By contrast, CRP and IL-6 did not correlate significantly with IL-6 sR. However, significant correlation was noted between IL-6 sR and sgp130.

**Conclusions:**

Concentrations of IL-6 and its soluble receptors were significantly elevated in patients with CSU, suggesting upregulation of the IL-6 trans-signaling in the disease. In addition, our results support the concept that the system may be involved in pathogenesis of the systemic inflammatory activation in CSU patients.

## Introduction

Chronic spontaneous urticaria (CSU) is a mast cell-dependent immuno-inflammatory disease, accompanied by different changes in the neuro-endocrine-immune system function, coagulation/fibrinolytic activity, and metabolic processes [1–4].

C-reactive protein (CRP) is a marker of CSU activity, reflecting the systemic effects of inflammatory mediators, mainly IL-6 [2,5,6]. IL-6 plays a pivotal role in immune and inflammatory responses and its effects are mediated through two different ways: the classical receptor signaling and the trans-signaling pathway. The biological activity of the trans-signaling pathway depends on IL-6, its soluble receptors subunit (IL-6 sR) and signal-transducing membrane glycoprotein 130 (gp130), which is widely expressed by most cell types [7–9].

The gp130 / IL-6 /IL-6 sR complex is critically involved in the promotion of several inflammatory and autoimmune diseases [8, 9]. In circulation IL-6 sR augments the activity of IL-6 trans-signaling, whereas soluble gp130 (sgp130) acts as an inhibitor, blocking IL-6/sIL-6 sR complex [7, 10].

The clinical relevance of the pathway in the pathogenesis of CSU is incompletely understood.

Recently, it has been reported that IL-6 family of cytokines, may play an important role in pathogenesis of CSU [2, 11].

To our knowledge, this has been the first attempt to provide a novel insight into the role of different components of IL-6 pathway in relationship between the disease activity and acute phase response in CSU. Therefore, we studied plasma concentrations of IL-6 and its soluble forms of receptors. In addition the correlations between IL-6, IL-6 sR, sgp130 and CRP concentrations were investigated.

## Materials and Methods

58 patients with active CSU (18 men and 40 women; median age: 38 years, range: 24–52) with a median disease duration of 3 years were enrolled in the study.

In all cases, any known causes of CSU were ruled out by appropriate investigations. Each patient underwent the following tests: routine laboratory tests (full blood count, urine analysis, ESR, C-reactive protein, serum glucose, hepatic functions, and creatinine), stool (for parasites), hepatitis serology, antinuclear and antithyroid microsomal antibodies, thyroid function, chest X-ray and abdominal ultrasonography. Additionally, dental, and ENT consultations as well as the autologous serum skin test (ASST) were performed [1].

UAS according to EAACI/GALEN/EDF guidelines [12] was estimated during four days and on the blood sampling day and graded as follows: mild (0–8), moderate (9–16) and severe (17–24). The study comprised 32 patients with mild and 26 patients with moderate-severe urticaria symptoms.

H1- antihistamine drugs were withdrawn at least 4 days before blood sampling. None of the patients had been taking immunosuppressants or any other drugs, for at least 8 weeks before the study.

The control group comprised 22 sex-, age- and BMI (< 30) matched the healthy subjects.

The Ethics Committee of the Medical University of Silesia (KNW-640-2-1-004/15) approved of the study and written, informed consent was obtained from all the subjects participating.

### Blood collection

Blood samples were taken on fasting, from elbow veins using tubes with anticoagulant. Plasma obtained by centrifugation were stored at -85°C until the tests were performed.

### Assay of Il-6, Il-6 sR and sgp130

Concentrations of Il-6, Il-6 sR and sgp130 in plasma samples were measured by ELISA method using commercially available kits (Quantikine ELISA from R&D Systems, Inc., MN, USA) according to manufacturers’ detailed instructions. The coefficients of variance for intra-assay and inter-assay were below 8%.

### CRP assay

Serum C-reactive protein (CRP) concentrations were assayed using Roche/Hitachi cobas c system. Normal lab ranges: below 5.0 mg/L.

### Statistical analysis

The obtained results were presented as basic parameters of descriptive statistics. Normal distribution of data was measured using Shapiro-Wilk’s test. Independent data between the groups of patients with CSU and the control group and between CSU patients with mild and moderate-severe symptoms were compared using non-parametric U Mann—Whitney test. The Spearman’s rank test was used for correlations. The p<0.05 was considered statistically significant. Calculations were performed with STATISTICA for Windows 10.0 software (StatSoft, Cracow, Poland).

## Results

### Plasma IL-6 concentration

**Fig 1:**
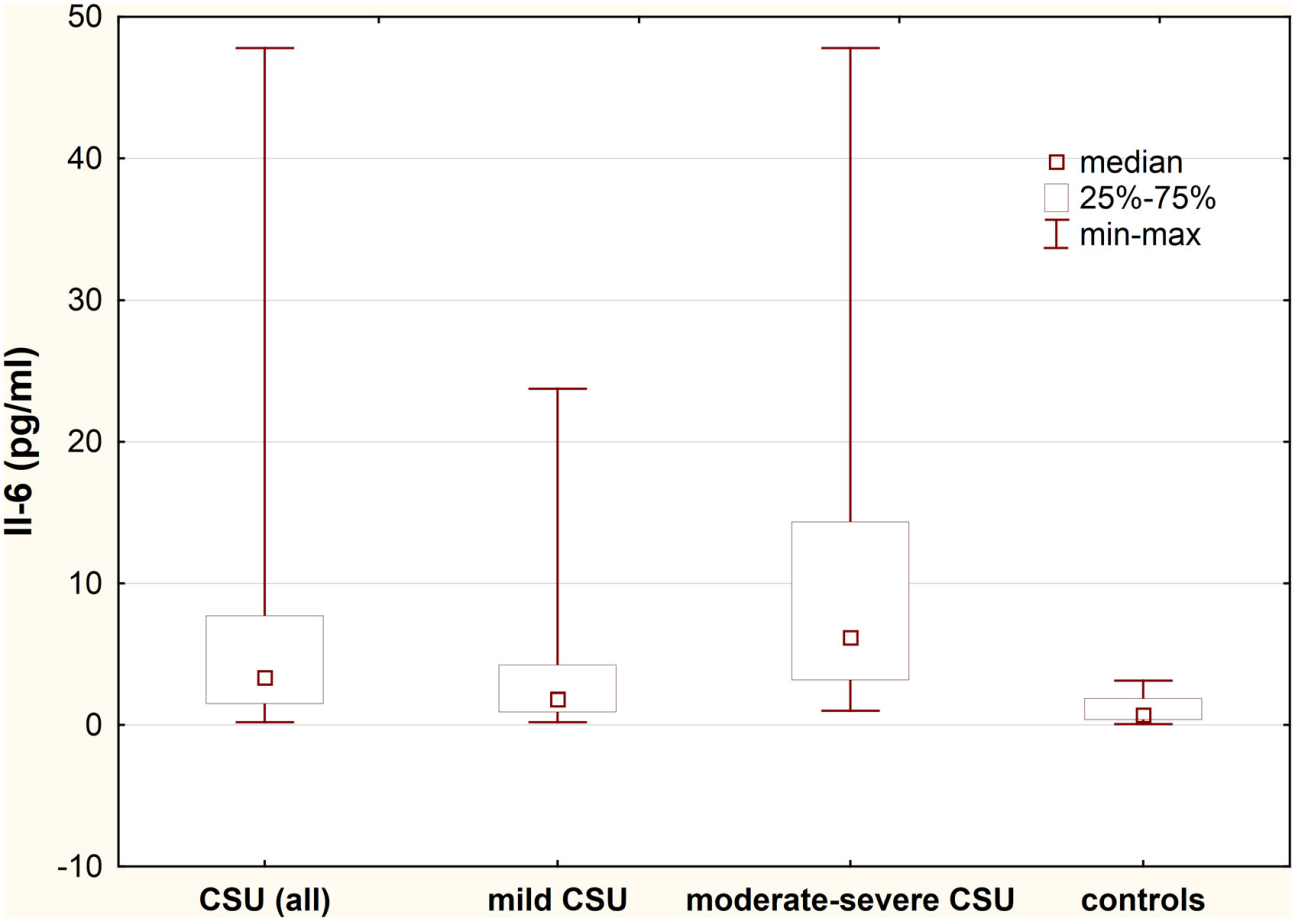
Plasma IL-6 concentration in chronic spontaneous urticaria (CSU) patients with different disease activity. CSU vs. controls, p<0.0001; moderate-severe CSU patients vs. mild CSU vs. controls, p<0.001 and p<0.0001, respectively; mild CSU vs. controls, p<0.001.

Plasma concentration of IL-6 was significantly higher in CSU patients as compared with the healthy subjects (median: 3.32 vs. 0.69 pg/ml; p<0.0001) (Fig 1).

IL-6 plasma concentration was significantly higher in moderate-severe CSU patients as compared with those with mild CSU and the healthy subjects (median: 6.16 *vs*. 1.82 *vs*. 0.69 pg/ml, p<0.001 and p<0.0001, respectively). In addition, plasma IL-6 concentration in mild CSU patients was significantly higher than in the health subject (1.82 vs. 0.69 pg/ml, p<0.001).

No significant differences in IL-6 concentrations between ASST(+) and ASST(-) CSU patients (selected according to the similar UAS) were observed.

### Plasma IL-6 sR concentration

**Fig 2:**
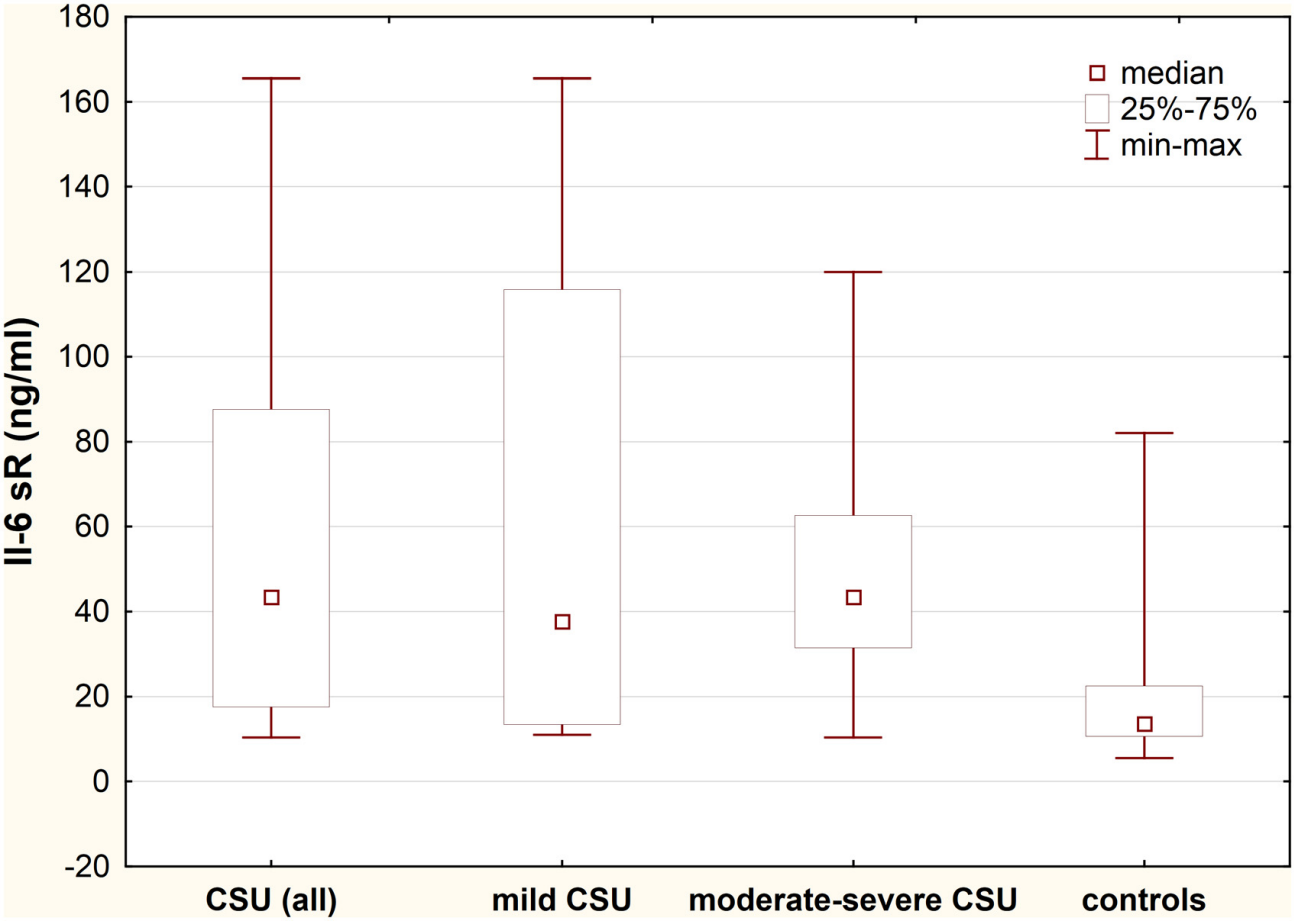
Plasma IL-6 sR concentration in chronic spontaneous urticaria (CSU) patients with different disease activity. CSU vs. controls, p<0.0001; moderate-severe CSU patients vs. mild CSU vs. controls, p>0.05 and p<0.0001, respectively; mild CSU vs. controls, p<0.01.

Plasma concentration of IL-6 sR was significantly higher in CSU patients as compared with the healthy subjects (median: 43.38 vs. 13.6 ng/ml; p<0.0001) (Fig 2).

IL-6 sR plasma concentration was significantly higher in moderate-severe CSU patients as compared with the healthy subjects, but not with mild CSU patients (median: 43.38 *vs*. 13.6 *vs*. 37.62 ng/ml, p<0.0001 and p>0.05, respectively). However, plasma IL-6 sR concentration in mild CSU patients was significantly higher than in the health subjects (37.62 vs. 13.6 ng/ml, p<0.01).

No significant differences in IL-6 sR concentrations between ASST(+) and ASST(-) CSU patients (selected according to the similar UAS) were observed.

### Plasma sgp130 concentration

**Fig 3:**
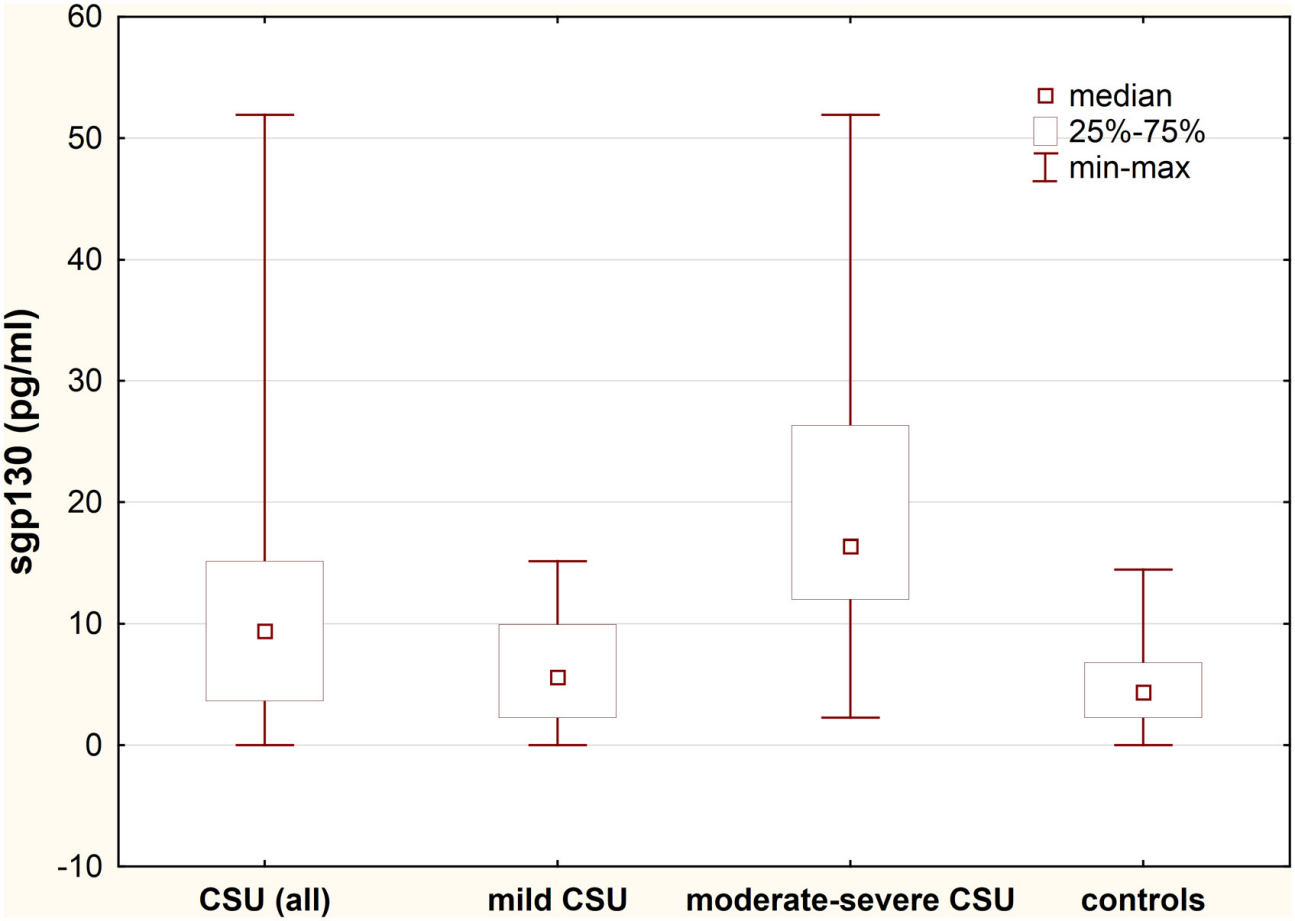
Plasma sgp130 concentration in chronic spontaneous urticaria (CSU) patients with different disease activity. CSU vs. controls, p<0.01; moderate-severe CSU patients vs. mild CSU vs. controls, p<0.0001; mild CSU vs. controls, p>0.05.

Plasma concentration of sgp130 was significantly higher in CSU patients as compared with the healthy subjects (median: 9.4 vs. 4.35 pg/ml; p<0.01) (Fig 3).

sgp130 plasma concentration was significantly higher in moderate-severe CSU patients as compared with those with mild CSU and the healthy subjects (median: 16.37 *vs*. 5.57 *vs*. 4.35 pg/ml, p<0.0001 and p<0.0001, respectively).

Concentration of sgp130 in mild CSU group did not differ significantly as compared with the healthy subject (median: 5.57 *vs*. 4.35 pg/ml, p>0.05) (Fig 3).

### Serum CRP concentration

**Fig 4:**
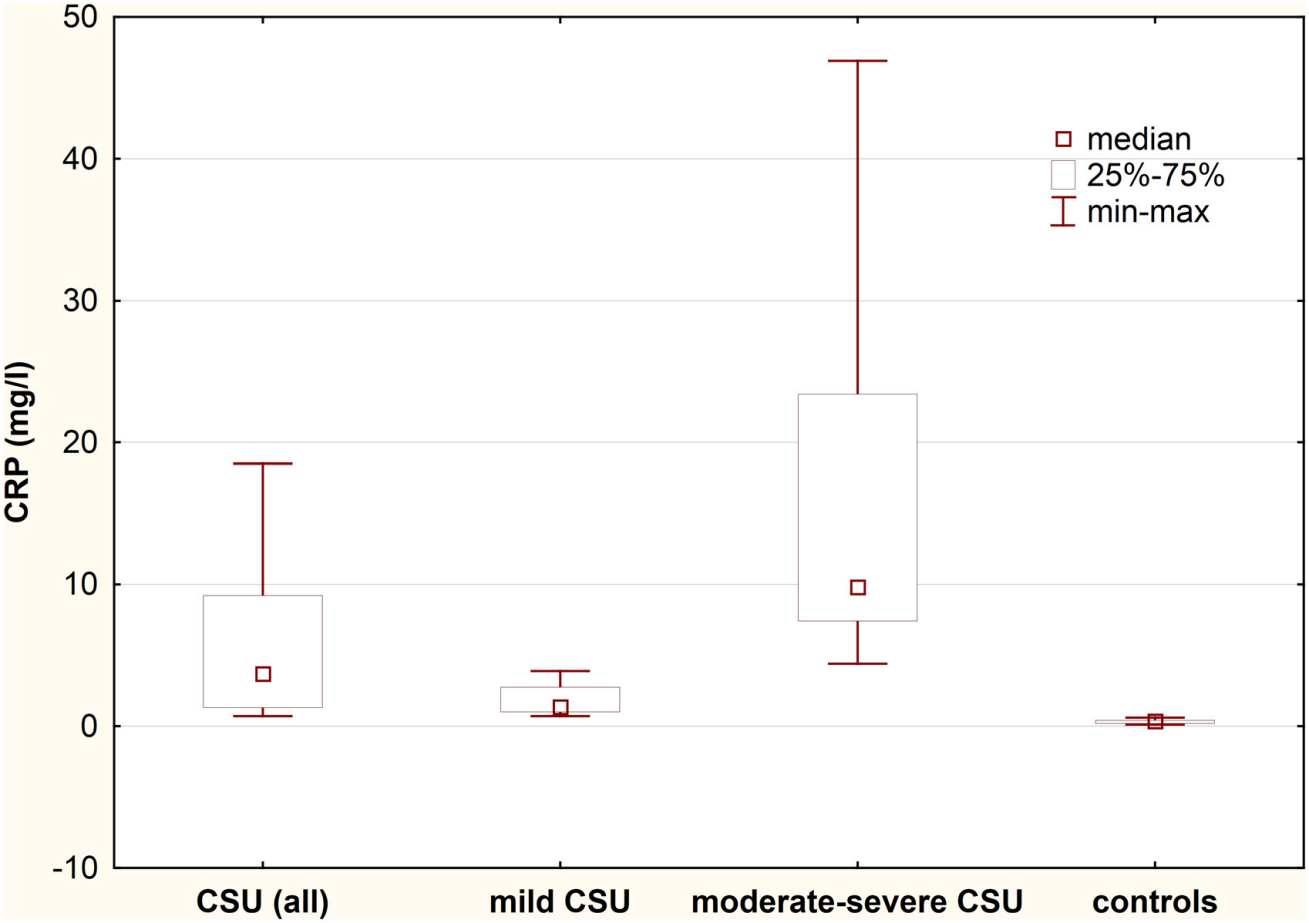
Serum CRP concentration in chronic spontaneous urticaria (CSU) patients with different disease activity. CSU vs. controls, p<0.0001; mild CSU vs. moderate-severe CSU vs. controls, p<0.001.

Serum CRP concentration was significantly higher in CSU patients as compared with the healthy subjects (median: 3.7 vs. 0.35 mg/l; p<0.0001). In addition, there were significant differences in serum CRP concentration between CSU patients with mild, moderate- severe symptoms and the healthy subjects (median: 1.35 vs. 9.8 vs. 0.35 mg/l, respectively; p<0.001) (Fig 4).

### Correlation between values of CRP, IL-6, IL-6 sR, and sgp130 in patients with CSU

**Fig 5:**
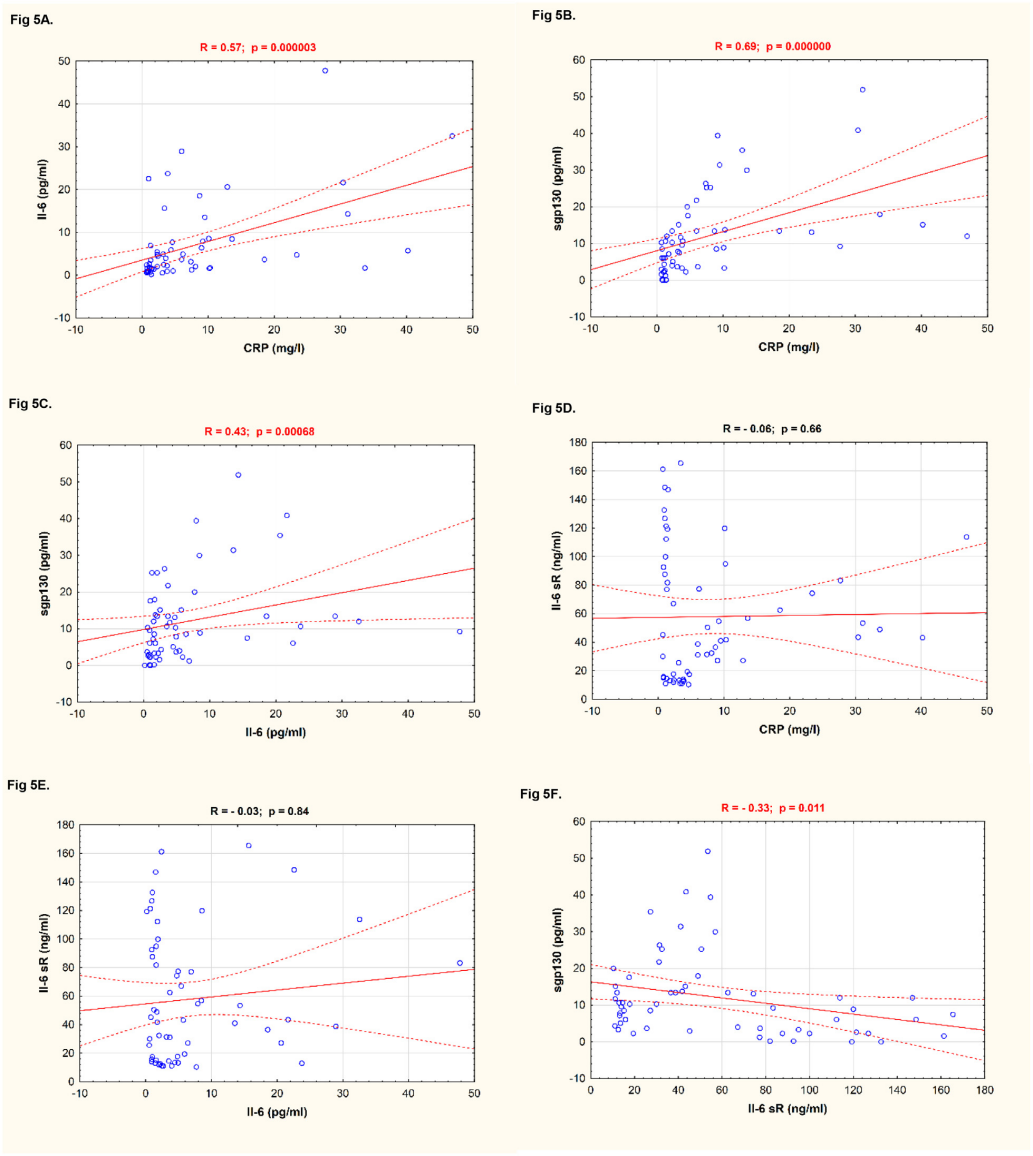
Correlation between components of IL-6 pathway and CRP in CSU patients. A. CRP vs. IL-6; B. CRP vs. sgp130; C. IL-6 vs. sgp130; D. CRP vs. IL-6 sR; E. IL-6 vs. IL-6 sR; F. IL-6 sR vs. sgp130.

CRP significantly correlated with IL-6 and sgp130, similarly IL-6 was significantly correlated with sgp130 (Fig 5A–5C). By contrast, CRP and IL-6 did not significantly correlate with IL-6 sR (Fig 5D and 5E). However, significant correlation was noted between IL-6 sR and sgp130 (Fig 5F).

## Discussion

The current study supports the thesis, that circulating concentrations of IL-6 and CRP increase in CSU patients [2]. There was significant positive correlation between CRP and IL-6, which confirms that IL-6 is associated with the regulation of CRP synthesis.

This has been the first investigation to demonstrate that alongside IL-6, CSU is associated with increased plasma concentrations of its both soluble receptors.

The effect of the cytokine depends on receptors that are released as soluble molecules; their concentrations are >1000-fold higher than IL-6 itself [13]. Therefore, in the present study, we focused on the circulating levels of IL-6 sR and sgp130, which have different functions.

In circulation elevated IL-6 sR acts to augment the activity of IL-6 leading to a greater IL-6/IL-6 sR complex formation, which can stimulate a great diversity of cells and is involved in many biological processes, including enhanced expression of proinflammatory mediators [10, 14]. Plasma concentration of IL-6 sR was significantly higher in CSU patients as compared with the healthy subjects. The presence of elevated IL-6 sR and IL-6 concentrations in CSU suggests that IL-6 acting via IL-6 sR enhances the trans-signaling capacity as a part of the inflammatory response and consequently may enhance the disease activity. It has been suggested, that IL-6 trans-signaling is critically involved in the maintenance of numerous clinical conditions, by promoting chronic inflammation [8].

It has been suggested that sleep together with circadian rhythm may enhance IL-6-mediated processes, which are associated with increased concentrations of IL-6 and IL-6 sR [13]. It is interesting to speculate that similar increased formation of IL-6/IL-6 sR complexes might occur during urticarial inflammation, which could partly account for the clinical symptoms of the disease frequently worsening at night, when physiological concentrations of IL-6 and IL-6 sR increase.

CSU patients show significant increase not only in plasma IL-6 and IL-6 sR concentrations, but also in sgp130, which acts as a negative regulator of IL-6 trans-signaling. Sgp130 inhibits binding of IL-6/IL-6 sR complex to membrane gp130, thus preventing activation of the biological activity of IL-6 [7, 8]. This is the first study to report elevated sgp130 plasma concentration of CSU patients with more severe disease activity. Up-regulation of sgp130 in CSU patients might suggest a compensatory mechanism, which controls intracellular IL-6 signaling and prevents the effects of the IL-6/IL-6 sR complexes, which may promote progression of the inflammatory disease [8–10].

Correlation observed between circulating concentrations of sgp130 and IL-6 sR suggests that these two types of receptors are regulated by the same mechanism in CSU.

In addition, there were significant correlations between concentrations of sgp130 and IL-6 and CRP in CSU patients. These findings suggest that sgp130 might be involved in regulation of the systemic inflammatory processes associated with both, classical receptor signaling and trans-signalling pathway.

The role of IL-6 trans-signaling in CSU remains unclear. The biological significance of IL-6 sR and its systemic or local sources have not been fully understood. It has been demonstrated that IL-6 sR may be released from activated leukocytes [8, 15]. The processes responsible for generation of sgp130 are still unknown and so we are unable to indicate mechanisms underlying such increased concentration of sgp130 in urticarial processes [14].

## Conclusions

Current data demonstrate that in the course of CSU plasma concentrations of IL-6 and its soluble receptors increase concurrent with elevated serum concentration of CRP. These results suggest that elevated plasma concentrations of the components may reflect activation of IL-6 trans-signaling system and may be accompanied by enhanced activation of acute phase response and the disease activity. However, further studies are needed to determine the sources of such increased circulating concentrations of IL-6, sIL-6 sR and sgp130.
